# IMP3 protein is an independent prognostic factor of clinical stage II rectal cancer

**DOI:** 10.1038/s41598-021-90513-y

**Published:** 2021-05-25

**Authors:** Daniela Bevanda Glibo, Danijel Bevanda, Katarina Vukojević, Snježana Tomić

**Affiliations:** 1 Department of Internal Medicine, University Hospital Center Mostar, 88000 Mostar, Bosnia and Herzegovina; 2 Department of Anatomy, Histology and Embryology, University of Split School of Medicine, Šoltanska 2, 21000 Split, Croatia; 3 Department of Medical Genetics, School of Medicine, University of Mostar, 88000 Mostar, Bosnia and Herzegovina; 4 Department of Pathology, Citology and Forensic Medicine, University Hospital Center Split, 21000 Split, Croatia

**Keywords:** Cancer, Gastroenterology, Oncology

## Abstract

Immunohistochemical level of IMP3-protein in patients with rectal cancer in clinical stage II (141), were correlated with sociodemographic, pathohistological and clinical indicators and duration of overall-survival and progression-free-survival. Vascular invasion was associated with IMP3-positive immunostaining (p < 0.001). Vascular invasion ratio in the group of poorly-differentiated-tumors was 21 times higher than in the group of well-differentiated-tumors. IMP3-positive patients lived 2.2 times shorter than negative (p < 0.001). Patients with well-differentiated-tumors lived 1.7 times longer than the subjects with poorly-differentiated-tumors (p < 0.001). Patients without vascular invasion lived 2.7 times longer than the subjects with vascular invasion (p < 0.001). The risk of mortality was 2.3 times higher for IMP3 positive patients (p = 0.027) and 10.4 higher for the patients with vascular invasion (p < 0.001). IMP3-negative participants had 2.3 times longer free interval without disease (p < 0.001). The free interval without disease was 3.6 times longer in the group without vascular invasion (p < 0.001). The risk of disease relapse in the IMP3 positive group was 5.3 times higher (p < 0.001) and with vascular invasion was 8 times longer (p < 0.001). The risk of disease relapse was 6.8 times higher in the group with vascular invasion (p < 0.001). Patients with rectal cancer and high IMP3-protein level will have a shorter overall survival relative to patients without or with low levels of IMP3. The analysis of IMP3 expression by immunohistochemistry pointed IMP3 as an independent prognostic factor of clinical stage II rectal cancer.

## Introduction

Colorectal cancer (CRC) is one of the most common malignant tumors worldwide, ranked as third malignancy in men (746,000 new cases per year), and as second in women (614,000 new cancer cases per year)^1^. A particularly high incidence was found in Western Europe, the United States and Australia, while the lowest incidence was in West Africa.

Due to high incidence and mortality, CRC represents a significant public health issue. Epidemiological and experimental studies as well as migration studies clearly point out the fact that environmental factors play a crucial role in the emergence of this disease.

Surgical treatment provides the only possibility of cure. Chemotherapy and radiotherapy are used to treat locally advanced and metastatic diseases. In assessing the use of adjuvant therapy, the most important parameter is the clinical stage of the disease^2^.

In patients with clinical stage II, chemotherapy is indicated individually. A large number of researchers are trying to find additional indicators that would select subgroup of patients in stage II colorectal cancer which could have benefit from the chemotherapy^3, 4^. Tumor invasiveness and its metastatic potential are the main determinants of outcomes of CRC, and factors involved in these processes are obvious candidates for new prognostic indicators^5^. One of these is IMP3 protein. IMP3 belongs to the family of insulin-like growth factor II mRNA binding proteins (IMPs) that play a key role in the transmission and stabilization of RNA, cell growth and migration during embryogenesis^6^. IMP3 also plays an important role in carcinogenesis and progression of colon cancer and can serve as a prognostic biomarker to identify patients with a risk of developing metastasis or recurrence of cancer after the colectomy. So far, IMP3 has been investigated in various malignant tumors. Studies have shown that IMP3 expression is negative in healthy mature tissue and is positive in malignant neoplasms of the colon, kidney, bladder, pancreas, stomach, breast and lung, and is associated with an advanced clinical stage with distant metastases and shorter overall survival^7–10^.

There are only five published studies in which IMP3 immunoexpression in colorectal cancer were analysed^4, 11, 12^. In this study, IMP3 expression was analyzed for the first time in tumor samples of a selected population with rectal cancer in clinical stage II.

## Materials and methods

### Data collection

The Ethical Committee of University Hospital Center (UHC) Split approved the study. The study was conducted according to the guidelines of the Declaration of Helsinki, Patient Rights Protection Act (NN 169/04), Law on the Implementation of the General Regulation on Data Protection (NN42/18), Croatian Code of Medical Ethics and Deontology (NN55/08, 139/15). All patients have agreed to participate and have signed Informed Consent. The study was conducted on samples of paraffin-embedded tumor tissue, taken from patients diagnosed with clinical stage II rectal cancer during the period from 1 January 2005 to 31 December 2007. All patients were operated at the Department of Surgery of UHC Split. The exclusion criteria were: patients who had only a palliative surgical procedure, patients who received radiotherapy or/and chemotherapy before surgery, patients with less than 12 lymph nodes examined in the pathohistological report, patients who did not had paraffin blocks of CRC in the archives of the Department of Pathology and patients who were not monitored at Department of Oncology and Radiotherapy of UHC Split.

Socio-demographic and clinical data have been collected from the initial operating protocol and the history of the disease: age, gender, operation date, tumor size and clinical stage. Patients with CRC were divided into age groups in four categories: younger than 25, 25 to 44, 45 to 64 and over 65.

Pathohistological data were collected from the pathohistological report: pathological stage, tumor size, histological type, degree of differentiation, vascular invasion. The clinical stage of the tumor was determined according to the TNM classification. Tumor stage II is classified as T3 N0 M0^13, 14^. The degree of differentiation was determined by the percentage of glandular and solid components. Tumors with less than 50% of the solid component were classified in the low-grade group and more than 50% of the solid component in the high-grade group^15^. Positive vascular invasion was defined as finding a tumor embolus within the endothelium lined spaces on the invasive edges of the tumor.

Data on survival, recurrence and duration of disease free survival were obtained from files of patients treated at the Department of Oncology and Radiotherapy and Department of Surgery of UHC Split. The period of disease length monitoring was considered as the time interval between the date of surgery and the date of the last clinical checkup or death of the patient (until 31/12/2012). The free interval without recurrence of the disease was considered as the time interval between the date of surgery and the date of the recurrence of diagnosis (until 31/12/2012).

### Immunohistochemistry

**Figure 1:**
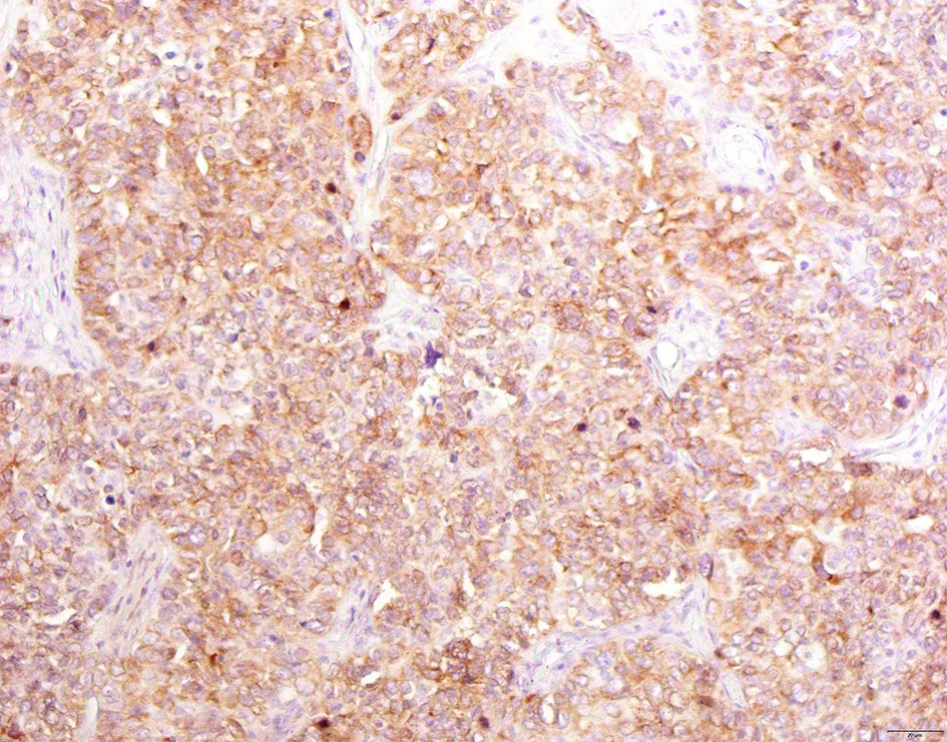
Immunohistochemical staining with IMP3 in patients with rectal cancer in clinical stage II. Positive cells display brown cytoplasmic staining.

Representative tumor tissue sections were stained using the method of immunohistochemistry in the Laboratory for Immunohistochemistry of the Department of Pathology, Forensic Medicine and Cytology, UHC Split. Immunohistochemical analysis was performed on 5 μm sections using primary monoclonal mouse antibodies against IMP3 (dilution 1:100, M3626, clone 69.1), EnVision/HRP and chromogen 3,3'-diaminobenzidine (all reagents DAKO, Glostrup), on DAKO automatic immunostainer. Two experienced pathologist analysed the slides with light microscope Olympus 51BX. Diffuse cytoplasmic staining for IMP3 with more than 10% of positive cells were considered as positive (Fig. 1).

### Statistical analysis

The data were processed in the SPSS 17 software and descriptive statistics methods were used. The data were tested by Kolgomorov-Smirnov test. The correlation of qualitative variables with the investigated groups was determined by χ2 test and binary logistic regression (OR). The Mann–Whitney test and the Kruskal–Wallis test were used to test the differences in quantitative variables between the investigated groups. In the analysis of survival and disease recurrence, the Log rank test, Kaplan Meier survival curves and Cox regression analysis were used. The results were interpreted at the significance level p < 0.05.

### Ethics approval and consent to participate

The Ethical Committee of University Hospital Center (UHC) Split approved the study.

## Results

The study included 141 subjects with diagnosis of clinical stage II rectal cancer with median of 62 years of age (min–max 39–79 years) (Table 1). In the group of patients that died of rectal cancer there were 3.9 times more IMP3 positive expression than in the group of survived patients (p < 0.001). In the group of patients that died of cancer there were 7 times more poorly differentiated carcinoma than in the group of survived patients (p < 0.001). In the group of patients that died of cancer there were 27 times more findings of vascular invasion than in the group of survived patients (p < 0.001) (Table 1).

**Table 1:** The number (%) of patients with CRC and clinical stage II compared to the investigated parameters or median (min–max) of the patients compared to the outcome of the disease.

	Total	Outbreak of illness			P*
		Died from cancer	Another cause	Alive	
**Gender**					
Male	85 (62)	46 (70)	9	30 (52)	0.103*
Female	52 (38)	20 (30)	4	28 (48)	
Age	62 (39–79)	65 (45–79)	70 (39–75)	60 (42–74)	0.005**
Tumor size (cm)	5 (2–17)	4.5 (2–10)	6 (4–17)	5 (2–12)	< 0.001**
**IMP3**					
Negative	63 (46)	12 (18)	5	46 (79)	< 0.001*
Positive	74 (54)	54 (82)	8	12 (21)	
**Degree of differentiation**					
Well	99 (72)	34 (519)	11	54 (93)	< 0.001*
Poor	38 (28)	32 (49)	2	4 (7)	
**Vascular invasion**					
No	79 (589)	12 (18)	11	56 (97)	< 0.001*
Yes	58 (42)	54 (82)	2	2 (3)	
**Age**					
≤ 62	68 (50)	29 (44)	4	35 (60)	0.007*
> 62	69 (50)	37 (56)	9	23 (40)	
**Tumor size (cm)**					
≤ 5	82 (60)	45 (68)	3	34 (59)	0.10*
> 5	55 (40)	21 (32)	10	24 (41)	

*χ2 test; †Mann–Whitney U test; ** Kruskal–Wallis test.

Based on the median age we divided the subjects into two categories (≤ 62 years, > 62 years). However, we did not demonstrate a statistically significant difference in the distribution of subjects by age groups relative to the outcome of the disease (χ2 = 5.4, p = 0.68).

Based on the median tumor size we divided the subjects into two groups (≤ 5 cm, > 5 cm). There was 1.9 times higher tumor size (more than 5 cm) in the group of patients who died of other causes compared to survived group of patients. Additionally, there was 2.4 times higher rate of tumor size more than 5 cm, in the group of patients that died of cancer compared to survived group of patients (p = 0.010) (Table 1). There was no statistically significant gender correlation with IMP3 expression (p = 0.075). We have not demonstrated statistically significant association of age groups with IMP3 expression (p = 0.971). There was no statistically significant association of tumor size with IMP3 expression (p = 0.146). In the group of patients with IMP3 positive expression there were 2.5 times more the poorly differentiated tumors compared to group of patients with IMP3 negative expression (p = 0.008).

In the group of patients with IMP3 positive expression there were 4.2 times more vascular invasion than in the group of patients with IMP3 negative expression (p < 0.001) (Table 2).

**Table 2:** The number (%) of patients according to the examined variables and logistic regression results in relation to IMP3.

	IMP3			OR 95%CI	P†
	Negative	Positive	P*		
**Gender**					
Male	34 (52)	52 (68)	0.075		
Female	31 (48)	24 (32)			
**Age**					
≤ 62	34 (52)	38 (50)	0.971		
> 62	31 (48)	38 (50)			
**Tumor size**					
≤ 5	43 (66)	40 (53)	0.146		
> 5	22 (34)	36 (47)			
**Degree of differentiation**					
Well	55 (85)	48 (63)	0.008	3.2(1.4–7.3)	0.005
Poor	10 (15)	28 (37)			
**Vascular invasion**					
No	55 (85)	28 (37)	< 0.001	9.4 (4–21)	< 0.001
Yes	10 (15)	48 (63)			

*χ2test; ^†^Logistic regression.

Table 3 showed the survival analysis of the log rank disease free survival (DFS) test according to the investigated indicators. We did not find a statistically significant difference for the disease free survival (DFS) among a group of patients with a tumor size of ≤ 5 cm and a group of subjects with a tumor size > 5 cm (p = 0.435) (Table 4).

**Table 3:** Results of Cox's regression multinomial analysis for disease free survival (DFS).

	RR	95% CI	P
**Gender**			
Male	0.694	0.42–1.24	0.177
Female*			
**Age**			
≤ 62*	1.2	0.74–2.1	0.415
> 62			
**Tumor size**			
≤ 5			
> 5*	0.79	0.5–1.3	0.334
**IMP3**			
Negative*	2.7	1.4–5.1	0.003
Positive			
**Degree of differentiation**			
Well*	0.575	0.326–1.0	0.057
Poor			
**Vascular invasion**			
No*	6.8	3.5–13	< 0.001
Yes			

*Reference level.

**Table 4:** Results of the Log rank test for the disease-free interval (DFS) according to the examined indicators.

	Average DFS (months)	95%CI	Median (SE) (months)	95%CI	LR*	P
**Gender**						
Male	58 (5)	48–68	44 (7)	30–58	5.2	0.022
Female	77 (6)	64–89				
**Age**						
≤ 62	74 (5.5)	63–85			5.8	0.016
> 62	56 (5.6)	45–67	44 (7)	30–57		
**Tumor size**						
≤ 5	62 (5)	52–73	50 (4.5)	41–59	0.61	0.435
> 5	82 (6)	70–94				
**IMP3**						
Negative	94(5)	84–104				
Positive	41.5 (4.5)	33–50	24 (5)	14–34	44	< 0.001
**Degree of differentiation**						
Poor	76 (5)	66–85				
Well	38 (5)	28–48	28 (9)	9.8–46	21.5	< 0.001
**Vascular invasion**						
No	93 (4.5)	84–102				
Yes	26 (3)	20–32	17 (4)	8.5–26	86	< 0.001

*Log rank test.

**Figure 2:**
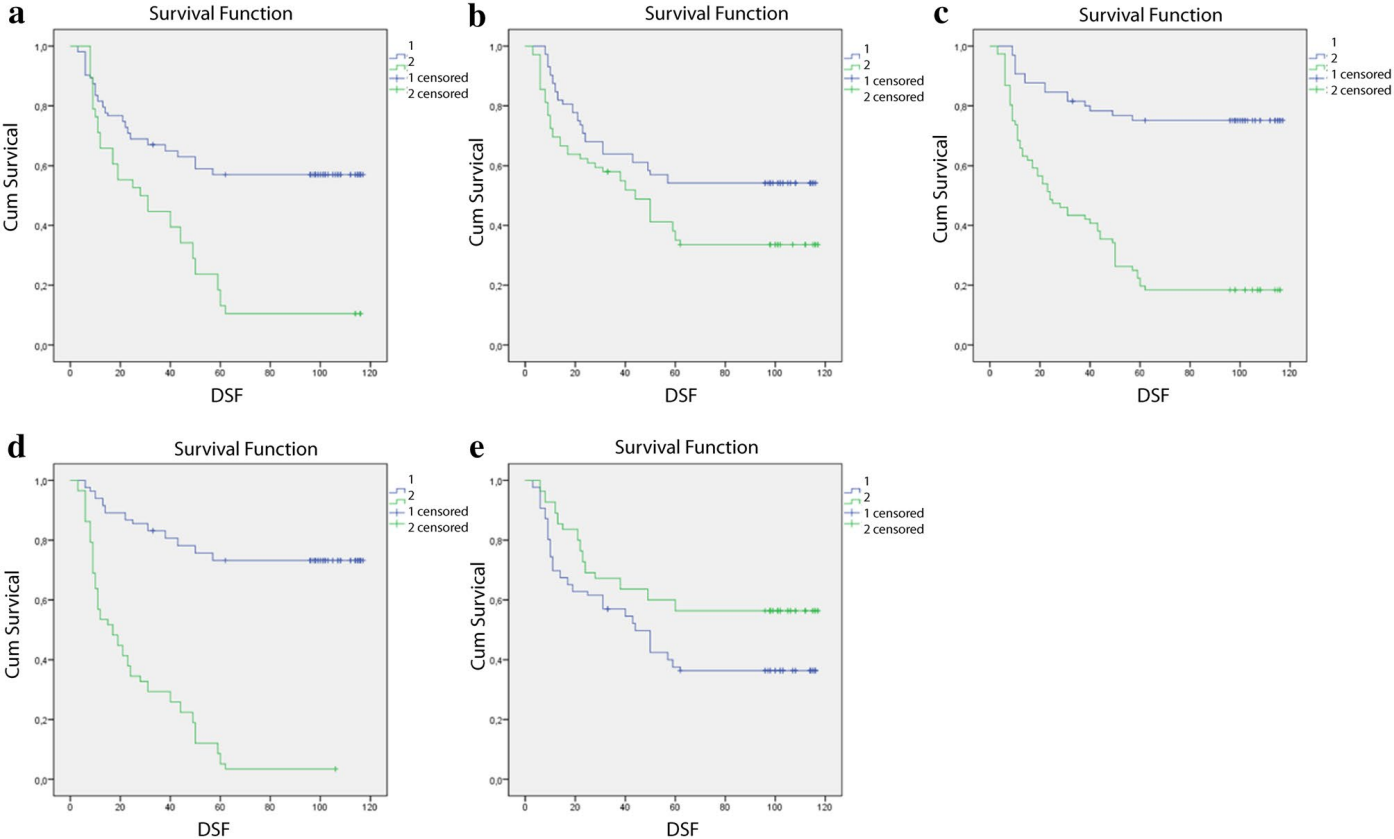
Kaplan–Meier disease free interval (DFS) curves relative to different indicators. (**a**) degree of differentiation: well differentiated carcinomas (blue line); poorly differentiated carcinomas (green line). (**b**) age groups: ≤ 62 (blue line); > 62 (green line). (**c**) expression of IMP 3: IMP 3 negative expression (blue line); IMP 3 positive expression (green line). (**d**) vascular invasion: no vascular invasion (blue line); vascular invasion present (green line). (**e**) gender: female (green line); male (blue line).

Disease free survival (DFS) in the group of subjects with negative IMP3 expression was 52.5 months longer than in the group of subjects with positive IMP3 expression (Fig. 2). Disease free survival (DFS) in the group of patients with well-differentiated tumor was 38 months longer than in the group of patients with poorly differentiated tumors. Disease free survival (DFS) was 67 months longer in the group of subjects without vascular invasion than in the group of subjects with vascular invasion (Table 2).

Multinomial regression Cox's analysis revealed that the risk of disease recurrence was 2.7 times higher in the group of subjects with positive IMP3 expression than in subjects with negative IMP3 expression (p = 0.003), and that the risk of disease recurrence was 6.8 times higher in the group of subjects with vascular invasion vs. group of subjects without vascular invasion (p < 0.001) (Table 3).

**Figure 3:**
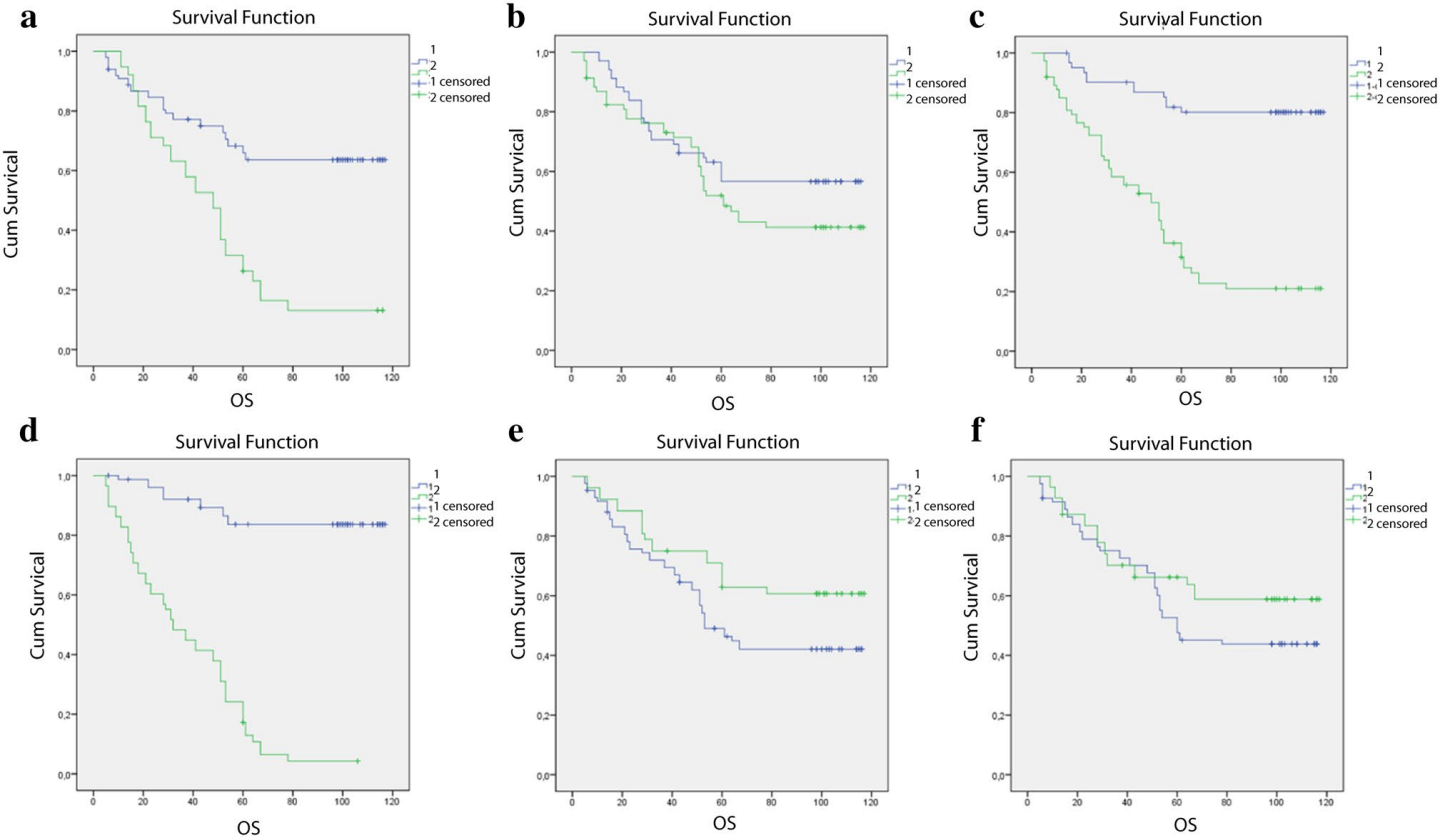
Kaplan–Meier's overall survival (OS) curve relative to different indicators. (**a**) degree of differentiation: well differentiated cancer (blue line); poorly differentiated cancer (green line). (**b**) age groups: ≤ 62 (blue line); > 62 (green line). (**c**) expression of IMP 3: IMP 3 negative expression (blue line); IMP 3 positive expression (green line). (**d**) vascular invasion: no vascular invasion (blue line); vascular invasion present (green line). (**e**) gender: female (green line); men (blue line). (**f**) tumor size: ≤ 5 cm (green line); > 5 cm (blue line).

The overall survival rate of women was 16 months longer than in men (p = 0.041). There was no statistically significant difference in survival among age group ≤ 62 and > 62 years (p = 0.136), nor in the survival length between subjects with tumor size ≤ 5 cm and > 5 cm (p = 0.166). Subjects with positive IMP3 expression lived 2.2 times shorter than subjects with negative IMP3 expression (p < 0.001) (Fig. 3). Subjects with well-differentiated tumors lived 1.7 times longer than subjects with poorly differentiated tumors (p < 0.001), while subjects without vascular invasion lived 2.7 times longer than subjects with vascular invasion (p < 0.001) (Table 5, Fig. 2).

**Table 5:** Results of analysis overall survival of respondents compared to researched variables.

	Average OS (SE) (months)	95%CI	Median(SE) (months)	95%CI	LR*	P
**Gender**						
Male	69 (5)	59–78	53 (6)	41–65	4.2	0.041
Female	85 (6)	74–96				
**Age**						
≤ 62	80 (5)	69–90			2.2	0.136
> 62	70 (5)	60–80	61 (6)	49–73		
**Tumor size**						
≤ 5	71 (5)	62–80	60 (12)	36–84	1.9	0.166
> 5	82 (6)	70–94				
**IMP3**						
Negative	100.6 (4)	92–109				
Positive	52 (4.5)	43–61	48 (7)	34–61	43	< 0.001
**Degree of differentiation**						
Well	86 (4)	77–94				
Poor	50 (5)	39–60	48 (5)	37–58	24	< 0.001
**Vascular invasion**						
No	104 (3.5)	97–111				
Yes	38 (3.3)	31–44	32 (5)	22–42	92	< 0.001

*Log rank test.

Multinominal regression Cox's analysis showed that the risk of death in CRC patients was 2.3 times higher in patients with IMP3 positive expression than in patients with IMP3 negative expression (p = 0.027), also there was 10.4 times higher mortality rate in subjects with vascular invasion compared to patients without vascular invasion (p < 0.001). We also found that risk of death for men was 1.7 times higher than for women at level of significance of 93% (p = 0.073) (Table 6).

**Table 6:** Results of Cox's regression multinomial analysis for overall survival of CRC clinical stage II.

	RR	95% CI	P
**Gender**			
Male	1.7	0.95–3.14	0.073
Female*			
**Age**			
≤ 62*	1.3	0.71–2.26	0.434
> 62			
**Tumor size**			
≤ 5			
> 5*	1.2	0.72–2.1	0.463
**IMP3**			
Negative*	2.26	1.1–4.6	0.027
Positive			
**Degree of differentiation**			
Well*	0.786	0.439–1.4	0.418
Poor			
**Vascular invasion**			
No*	10.4	4.6–2.3	< 0.001
Yes			

*Reference level.

The analysis of IMP3 immunohistochemical expression pointed IMP3 as an independent prognostic factor of clinical stage II rectal cancer.

## Discussion

Although colorectal cancer is one of the few cancers with early detection capability that allows for proper surgical removal, it is still among the malignant neoplasms in the third place by frequency, behind prostate and lung cancer in men, and secondly, behind breast cancer, in woman^13, 15^. Significant progress has been made over the last few years in understanding the molecular pathways of colorectal carcinogenesis, which enabled more specific targeting of colorectal cancer and improved screening programs for hereditary colorectal cancer. The treatment of this type of cancer is mostly surgical, and the decision on the use of adjuvant therapy depends primarily on the clinical stage of the disease^13, 15^. Although in most studies of colorectal cancer the clinical stage has proved to be the most important prognostic indicator, its clinical use is often insufficient to assess the need for additional treatment methods, especially in patients at clinical stage II^13^. Therefore, in this study, the expression of IMP3 protein in tissue samples of patients with rectal carcinomas was investigated in this prognostically and therapeutically the least defined clinical stage II.

In this study, using the method of immunohistochemistry, we investigated the expression of IMP3 protein, which plays a key role in the transmission and stabilization of RNA, cell growth and migration during embryogenesis. IMP3 has not been expressed in normal adult human cells. However, this cancer-specific gene is associated with different types of malignant tumors^16^. IMP3 is an mRNA-binding protein that function in RNA localization, stabilization and trafficking^17^. This protein exhibits the properties of an oncofetal protein, and its expression correlates with the aggressive behaviour of many tumors (lung cancer, renal cell cancer, oral cancer, urothelial carcinoma, hepatocellular carcinoma, pancreatic cancer, gastric cancer, and intrahepatic cholangiocarcinoma, as well as in the colorectal cancer) with adverse survival. Namely, it has been previously reported that IMP3 shows a positive correlation with clinical stage and degree of differentiation, increased number of local lymph nodes and distant metastases in advanced kidney, colon, bladder, pancreas, stomach, breast and lung cancer^4, 11^. Additionally, IMP3 was expressed in the reactivated stroma, in the prostate cancer, suggesting that IMP3 has influence in stromal‑epithelial interaction and fibroblast‑to‑myofibroblast differentiation^18^. Huang et al. observed IMP3 expression in tumor stroma cells in CRC associated with TNM stage, lymph node metastasis, lympho‑vascular invasion and tumor border^19^. This might indicate involvement of IMP3 in malignant progression and metastasis due to certain correlation between IMP3 expression and vascular invasion that we observed in our study. Prognostic value of IMP3 was investigated in relation to age, gender, histological type of tumor, degree of differentiation, vascular invasion, duration of overall survival (OS) and progression free survival (PFS). Our study included 141 patients with rectal cancer in clinical stage II with median age of 62 years. The median age of males is 8 years greater than in females, which is in accordance with other published study^4, 11, 19^.

Lockhead et al. analysed IMP3 immunoexpression in 212 patients with colorectal cancer in clinical stage II, but unlike our study, they used microarray technique and include patients with colon and rectal cancer^4^.

Huang et al. analysed IMP3 immunoexpression in 47 subjects in stage II, but in statistical analysis they merged the clinical stages I and II and concluded that IMP3 was mostly positive in the cells (62%) in relation to the stroma^19^.

Our results did not confirm the statistically significant association of sex, age groups and tumor size with IMP3. Contrary, to this finding Schulze et al. reported survival advantage of women for most, but not all cancer types^20^.

The difference in survival in relation to gender was found in only two studies. Prees et al. explained these differences by the fact that the colon mucosa has both estrogen and androgen receptors and that high expression of epidermal growth factor is associated (EGFR) with a worse prognosis especially in relation to both receptor types. Prees states that the EGFR activation pathway may be activated in a sexually specific manner, via androgenic and estrogenic receptors.

More than 50% of the survivors had a well-differentiated carcinoma, and in about 70% of the survivors there was no vascular invasion. Vascular invasion is also statistically significantly associated with expression of IMP3 and indicates greater invasiveness and metastatic ability of tumor cells by increasing cellular mobility and transendothelial migration in subjects with higher expression of IMP3. The correlation between vascular invasion and positive IMP3 expression was also found in other studies^4, 8, 11, 21^.

According to the results of the published studies, the high level of IMP3 immunoexpression of the IMP3 protein is associated with advanced clinical stage, distant metastasis and shorter overall survival and disease free survival (DFS)^11, 12, 19, 21^.

Contemporary oncology therapy should be tailored to the patient and molecular targeting of cancer. In addition, there is a possibility of immunotherapy in which vaccination against tumor-specific antigens and stimulation of a specific cellular response could contribute to a better survival of the patient. IMP3 has characteristics suitable for the development of such carcinoma vaccine because it is highly immunogenic, often expressed in carcinomas, particularly in malignant cells^22^. First clinical studies show that vaccination with IMP3 stimulates cytotoxic response in advanced opioid patients and has a beneficial effect on overall survival^22^.

Therefore, one of the challenges is the detection of molecules that predict the metastatic potential of the tumor. To date, there are a small number of papers analyzing the expression of IMP3 in the whole tissue samples of rectal cancer. In one of the published studies only colon cancers were analyzed^11^, and in the remaining 4 studies CRC were analysed^4, 12, 19, 23^. In all five studies, patients in all clinical stages were analyzed, and the immunohistochemical level of IMP3 protein in one study was performed on microarray (10).

Therefore, our research, which expand existing knowledge on the role of IMP3 protein in rectal cancer, can contribute to the understanding of the IMP3 cellular pathway involved in invasion and metastasis in the rectum carcinomas. This is particularly important for patients in clinical stage II where defining additional prognostic factors can help assess the need for additional oncological treatment after primary surgical tumor resection. IMP3 protein could represent such an additional prognostic factor in these patients contributing to development of new diagnostic and treatment strategies.

## Data Availability

The datasets used and/or analysed during the current study are available from the corresponding author on reasonable request.
